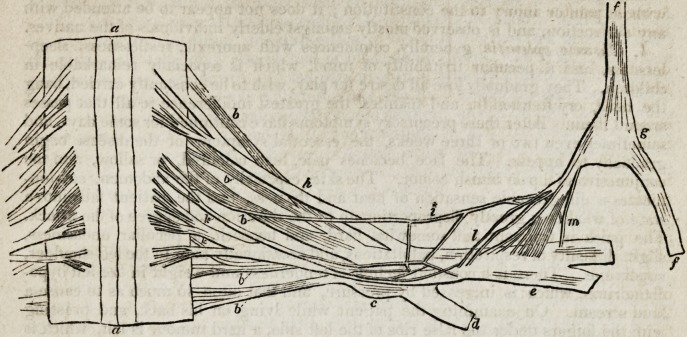# Physiology

**Published:** 1836-04

**Authors:** 


### PHYSIOLOGY.

### *Experiments on Saliva.* By Dr. C. H. SCHULTZ, Professor at the University of Berlin.

1. *Experiments on the Human Saliva.* Saliva was obtained for the purpose of these experiments, by Dr. S. and the students of his class exciting their salivary glands to an augmented secretion; which object was attained by taking hard cakes of sugar into their mouths, or by applying the tincture of the spilantha oleracea to their lips. Saliva thus collected, without the act of suction, is of course commixed with mucus from the mucous membrane of the mouth. It is translucid, tending very slightly to opacity, is considerably spumous, and ultimately deposits a cloudy sediment. It mostly contains alkali in a free state, in rare instances (during meals,) acid, and is now and then, during fasting, of a neutral character. In some cases a drop of vinegar scarcely suffices to neutralize a drachm of saliva; but whether thus neutralized or in itself originally neutral, nay, even if it at first contained free acid, it in every case assumes an alkaline character after remaining in a cool situation for sixteen or twenty-four hours, without evincing any traces of fermentation. The vapour emitted from alkaline saliva under the influence of heat restores the blue colour to reddened litmus paper, but the red colour returns if the paper be subsequently suspended in the air for a short space of time; and the same evanescent effect is produced upon reddened paper by recent fluid saliva. These circumstances prove the alkali to be volatile, in other words, *ammonia.* The extremity of a glass-rod, moistened with muriatic acid, soon exhibited acute white nebulæ of muriate of ammonia, when held over heated or even over cold saliva. Even in its dry state, the saliva still developed ammoniacal vapour, on carbonate of potass being superadded. Saliva does not coagulate from the influence of heat, a proof that it does not contain albumen: it forms a considerable scum at a temperature of 212° at first, but ceases or nearly ceases to do so after it has boiled for some little time.

2. *Experiments on the Saliva of the Horse.* A metallic tube communicating at its nether end with a bladder, was inserted into the divided Stenonian duct of a horse. For a whole day the parotid gland yielded no saliva, but a plentiful secretion ensued the next day, amounting, within twenty-four hours, to nearly fifty-six ounces; more than the half of which was obtained during the feeding hours. This saliva had the appearance of water slightly turbid, and slightly tinged with white wine. Its consistency was scarcely greater than that of water, and it frothed considerably on being shaken or poured from one vessel into another. Slender flocculi were seen suspended here and there in the fluid, but these afterwards collected at the bottom of the glass. Being separated from the fluid by filtration, they appeared to be convoluted membranous filaments, resembling epithelium. They were conceived by Dr. S. to be portions of the membrane lining the glandular ducts, and to have been elicited from thence by the corrosive tendency of the saliva. The specific gravity of the saliva was (at a temperature of 54° F.) 1.0125. Taste insipid, very slightly saline; chemical nature, powerfully alkaline, a grain of vinegar being required to neutralize a drachm of the saliva. Further experiments proved the alkali to be chiefly ammonia, although red paper, which had been restored to its original blue by the action of the saliva, was not again changed by exposure to the air. Saliva left standing for a week was found to have undergone decomposition, and to contain acetic acid in a disengaged state.

3. *Ptyaline.* Ten ounces of recent saliva obtained during feeding hours, and a like quantity procured during the hours of fasting, were separately evaporated to dryness, when each portion yielded a dry residuum of ninety grains, of a yellowish-white colour. This residuum rapidly attracted moisture from the atmosphere, and evinced the predominance of alkali.

The two portions of residuum, together 180 grains, were digested for six hours with seven ounces of alcohol, (spec. grav. 0.835,) by which means 110 grains were dissolved. The insoluble remainder, amounting in weight to seventy grains, was dryed, mixed with water and submitted to a gentle heat for six hours. A flocculent, greyish, yellow, diffuse deposit was thus produced, which being separated from the fluid by filtration, presented a doughy mass of the size of a walnut. Evaporated to dryness, it shrunk together, turned of a blackish grey, and weighed but eleven grains. The water had therefore dissolved fifty-nine grains. This solution, which according to Berzelius would contain the ptyaline, was of aqueous transparency, and did not froth like saliva. On its being dryed in a water bath, the entire exsiccated ptyaline did not, as Berzelius affirms, dissolve in water: on the contrary, an insoluble diffuse deposit was again formed.

4. *Examination of the Empty Stomach in Animals. First Experiment.* The stomach of a horse which, after fasting for several days, had died of tetanus, contained no remains of food beyond a single grain of oats. The fundus, however, or cul-de-sac (which in the horse was a lining of hard epidermis not extending to the pyloric portion), contained from three to four ounces of a turbid yellowish fluid, which after a time deposited a considerable flocculent sediment. This fluid quickly changed red paper to blue, as did likewise the coats of the stomach with which such paper was placed in contact.

Half an ounce of the above fluid was neutralized, under effervescence, by twenty-four drops of vinegar, and a series of experiments proved the alkali to be ammonia, whilst others showed some of the saline ingredients to be acetates. The deposit above described did not appear on further examination to contain any portion of bile, but to possess, on the other hand, a substance similar to that portion of saliva which is insoluble in alcohol.

The epidermis lining the fundus ventriculi exhibited isolated patches of erosion, a common phenomenon (according to Professor Gurlt, of the veterinary school at Berlin) with horses after a protracted fast. The author of the present experiments believes it to be owing to the powerfully alkaline nature of the fluid matter, and hints that spontaneous perforation of the coats of the stomach may arise from a similar cause.

*Second Experiment.* The stomach of a dog which was killed after fasting for forty hours, was found in a state of entire collapse, and its internal coat endued with a thick covering of mucus, in which alkali predominated. Water, in which, after the mucus had been entirely removed, the stomach was steeped for twelve hours, was found to have assumed a slimy consistency and to exert an alkaline reaction, although it had, notwithstanding, the property to curdle milk. In the jejunum of this dog a tænia cucurbitina was discovered, the greater part converted into a disorganized, pulpy mass—a proof, Dr. S. adds, that fasting has the power of causing intestinal worms to be digested.

The internal gastro-enteric membrane and the contents of the gall bladder were examined in a frog during the hibernating period, and found equally imbued with free alkali.

The experiments appear to succeed best when the test paper made use of is of a firm compact texture. When its texture is loose, the fluid to be tested is immediately imbibed, and the colours consequently become indistinct.

During the process of digestion, the acid of the chyme penetrates the whole parietes of the stomach, so that, at this period, the coats still retain an excess of acid even after having been steeped in water. This state continues for some time even after digestion has been completed; an uninterrupted influx of saliva, however, gradually neutralizes the acid, and alkali ultimately establishes itself in excess. Such a process of alternation, however, necessarily occupies a shorter or longer period (from eight to twenty-nine hours) according to the quantity of food to be digested. In the instances, therefore, where Professors Tiedemann and Gmelin observed acid to predominate in the stomachs of dogs which had been made to swallow flint pebbles, Dr. S. imagines that the experiments must have anticipated the neutralization consequent on digestion, as he considers it improbable that an excess of acid can have been the result of mere preternatural irritation.

Attributing the alkaline nature of the gastric contents (in the subjects of his own experiments) to the simple influx of saliva, Dr. S. explains the circumstance of fluid saliva being met with in the stomach of the horse and *not* in that of the dog, to the different constitution of the internal coat. In the horse, as already stated, the fundus ventriculi is armed with a stout epidermis which is very slightly endowed with the power of absorption or secretion—in the dog the more active membrane readily absorbs a large proportion of saliva, whilst it simultaneously secretes a quantity of mucus. Owing to this admixture, the contents of the dog's stomach when diluted with water will coagulate under the influence of heat,—a property denied to the fluid taken from the fundus ventriculi of the horse.—*Hecker's wissensch. Annalen, Zweit. B. Erst. Heft.* 1835.

---

*On the Respiration and Pulse of the Old.* By MM. HOURMANN and DECHAMBRE.

[IN No. I. page 233, we have given an analysis of the first part of this memoir, containing a description of the anatomical changes produced in the thorax by age. The second part embraces the modifications in the functions of the thoracic organs consequent on these changes. It must be recollected that two classes of aged females were alluded to, the first containing those who still retained their plumpness, and whose chests were large; the second, whose chests were contracted, and their whole appearance emaciated.]

1st Class. In an adult female the average admeasurement (by Baudelocque's pelvimeter) of the antero-posterior diameter of the chest during expiration is from six inches and a half to seven inches: the transverse, on a level with the nipple, is from nine and a half to ten inches; and towards the base, (level with the eighth rib) from eight inches and four lines, to nine inches: during a full inspiration the first diameter augments on an average one inch; the second one inch; the third one inch, eight lines. In old women of this class, the antero-posterior diameter during expiration is from seven to seven and a half inches; the superior and inferior transverse eight and a half to nine. The increase in inspiration is much less than in adults, rarely exceeding from four to five lines, and almost equal in the three directions. The sound on percussion is sonorous. When the lungs advance in front of the heart, the whole of the anterior part of the chest may give a clear sound: thus obscuring the diagnosis of diseases of the heart and pericardium; and simulating them if this portion of the lungs becomes indurated. When the liver is thrust down into the abdomen, and drags with it the right lung, the right side of the chest may give a clear sound to the same level as the left, or even lower. The respiratory murmur is distinctly vesicular, but larger, more noisy, less deep, and abundant.

2d Class. Emaciated old women, with contracted chests. As the respiratory organs become worn out, the necessity of employing them diminishes in a corresponding degree. Thus their movements are very unequal: both as to time and duration. During expiration the antero-posterior diameter is from seven to seven and a half inches: the superior transverse from eight to eight and a half; the inferior transverse from seven to seven and a half; in a full inspiration the first diameter increases from five to seven lines; the last two one, two, or three lines at most, and often not at all. Thus in old age the movements of the chest, particularly in the transverse direction, are considerably diminished. The elevation of the sternum is diminished, and the anterior projection of its inferior part is altogether prevented by the circular depression (previously mentioned), and the overlapping of the cartilages of the lower ribs, the effects of stays; as well as by the ossification of the cartilages of the first and second ribs. Owing to this diminished motion of the bony walls of the chest, the diaphragm often becomes the chief agent in inspiration; its action is redoubled, and its folds often form depressions on the posterior edge of the liver. The scaleni and sterno-cleido-mastoidei muscles are rendered useless where the spine is much curved; to remedy which the head is thrown back at each inspiration, so as to put these muscles on the stretch. Expiration is sudden and rapid, as if the whole bony framework fell as a single piece. Forced expiration, however, as in cough and expectoration, is difficult, for the recti, obliqui, and transversales abdominis are relaxed when the senile curvature is considerable.

*Percussion.* The sound on percussion is remarkably clear, equal to that of well-marked emphysema when the lungs are in the third type. The sound is modified by situation: thus the region corresponding to the inner half of the clavicle is much less sonorous than the anterior and superior region of the chest, which is owing to the almost constant presence of black or grey indurations of the upper part of the lungs, and to the very arched shape of the clavicle in the old. The sternal region is usually but little sonorous, owing to the diminished size of the lungs: the situation of the heart can therefore be exactly marked out by percussion.

*Auscultation.* Where the lungs are of the second type, respiration gives rather a blowing sound than a murmur; a sound like that produced by expelling the air through the compressed lips: and when the lungs are in the third type, this sound is so increased that it is more like general bronchial respiration. Its intensity is very variable: sometimes heard with difficulty, and the next moment noisy. When one lung is obstructed in any way, as in pneumonia, the respiratory murmur in the sound lung becomes more uniform, and in fact more vascular. The resonance of the voice is loud and noisy, almost bronchophonous: and in some so acute and interrupted as to become vibrating and jerking, as in ægophony.

*Relation between the State of the Pulse and the Respiration.* The examinations were made between half-past six and half-past seven in the morning, before the old women had eaten: some were in bed, others sitting on their beds. Three hundred and twelve were examined, 88 of whom were of the first class, and 214 of the second; 15 of the first class, and 42 of the second, were excluded, on account of the irregularity of the pulse: this irregularity was in the proportion of 1 to 6.53 for the first class, and 1.509 in the second; making a difference of 1.44. Consequently there was much less irregularity among those whose organization was most perfect. There remained 255 women, between the ages of sixty and ninety-six.

Sum of their ages 18,960; average, 74.33 years.
pulsations 20,984; 82.29 pulsations.
respirations 5,558; 21.79 respirations.

The relation consequently between the number of respirations and pulsations is as 1 to 3.41.

From this it appears that 82.29 is the mean number of pulsations of 255 aged women, contradicting the general opinion that the frequency of the pulse decreases with age. Thus Soemmering, Adelon, &c. give seventy pulsations as the average of the adult age, and sixty that of old age. MM. Leuret and Mitivié suspected this error from an examination of seventy-one of the aged inmates of the Bicêtre and Salpêtriere, and these researches on a larger scale confirm it. If, as M. Magendie supposes, there are twenty pulsations on an average every minute, in the adult, the frequency is increased in old age by 1.79; a proportion much less than the increase in pulsations. From comparison of the two classes of women another curious fact is obtained, confirming the preceding one.

Eighty-three of the 255 women, belonging to the first class.

Sum of ages 6195; average, 74.64 years.
pulsations 6673; — 80.42 pulsations.
respirations 1755; — 21.14 respirations.

172 belonging to the second class.

Sum of ages 12,765; average, 74.21 years.
pulsations 14,311; — 83.78 pulsations; an increase of 3.31 beyond the first class.

Sum of respirations 3803; average 22.11; or an increase of 00.97 beyond the first class.

The relation between the pulse and respiration hardly differs in the two classes.

Thus in the women of the first class who are younger in *organization*, though not in age, both the pulse and the respiration are less frequent than in the second class, who are worn out and decrepid; proving that senility is the real condition of this frequency.

It may be thought that some error has arisen from taking into the calculation those cases where the pulse was extremely frequent, although the individuals were in apparent health. To decide this point, a table is drawn up dividing the classes into six series, according to the rapidity of the pulse; from which it appears that in two-thirds the pulse was between seventy and eighty-nine, and in almost one-sixth between ninety and ninety-nine. Again, if those cases are excepted in which the pulse was below sixty and above eighty-nine, that is, very slow or very rapid, 185 individuals remain.

Their average age is 74.38
The average number of pulsations is 76.42
respirations is 20.91.

The relation between the respiration and pulse is 1 to 3.65. Thus after making the largest concessions, the frequency of the pulse is greater than, and that of respiration at least equal to, what is considered the average frequency in adults. In the following table the question is examined as to the relation between the respiration and pulsations, in those whose circulation and breathing were disordered. Out of the 255 there were 57 thus affected.

| | | |
|---|---|---|
| The average age of these 57 women is | | 66.50 |
| The average pulse | | 95.17 |
| | respirations | 27.75. |

Relation of the respiration to the pulse is 1 to 3.72, thus exceeding the average ratio in the whole number by 0.31; and proving the same relation, though less decidedly.

The commonly received opinion that the pulse decreases in frequency in old age is probably owing to instances occasionally occurring in which the pulse is remarkably slow. Thus these authors have felt it at thirty, twenty-nine, twenty-eight, in women whose only disease appeared to be senile marasmus. Generally in old age the pulse attains extreme degrees of slowness, or frequency, but the first is the exception and the second is the rule. The error has arisen from taking the one for the other.

[In a practical point of view the statement here made, that the pulse increases in frequency in old age, deserves much attention. Those who have opportunities of examining the truth of it for themselves, in extensive workhouses, should bear it in mind.]—*Archives Génerales de Médecine, Novembre,* 1835.

---

*Experiments on the Brain, Spinal Marrow, and Nerves. (With Woodcuts.)* By Professor Mayer, of Bonn.

Among the physiologists who have endeavoured to investigate the functions of the brain and nervous system in modern times, Professor Mayer places our distinguished countryman Sir C. Bell foremost on the list; adding the names of Rolando, Bellingeri, Magendie, and Flourens. The experiments of Flourens are some of the most important on this subject, but the great objection to them is the extensive injury which was unavoidably produced upon the brain, its nerves, and vessels, during these experiments; so that the precise effects upon the brain were probably more or less modified by the effects of the operations. Professor Mayer's object has been to repeat the experiments on a more simple plan; to avoid opening the cavity of the cranium, wounding the vessels, the severe hemorrhage, and exposure of the wounded parts to the atmospheric air; and thus to make the result more simple and certain. He has endeavoured to determine how far the influence which the activity of the brain exerts upon the organic system is disturbed, where, without having undergone any injury, its supply of blood, which we must look upon as the chief source of its vitality and activity, has been partly or wholly cut off.

We may effect this object in two ways: viz. by tying the arteries which supply the brain, and by injecting some foreign fluid into these vessels. With regard to the first method, this chiefly refers to putting a ligature upon the two carotids, because tying the vertebral arteries at the same time is not only almost impossible to effect, but, as we shall also find, is instantly fatal. On the other hand, putting a ligature upon both carotids, and also upon one subclavian, may be effected, as will be seen shortly.

The experiments of tying both carotids, besides their physiological value, are interesting to the medical practitioner, because cases might occur where it was necessary to undertake this operation in the human subject: it is therefore highly desirable to be previously acquainted with its effects on animals. This applies equally to the operation of tying one carotid.

"On a former occasion," says Professor Mayer, "I have called the attention of the surgical part of the profession to a consequence of tying the carotid, as regards morbid action in the eye.* These views have been recently confirmed by a new fact, viz. that in the operation for tying the carotid of a man, which was performed by Professor Bünger at Marburg, the eye of the same side suffered considerably."

It will be impossible to give a detailed account of these experiments; we shall therefore content ourselves with presenting to our readers a short summary, which the author himself has given at the end of the physiological part.

* Journ. für Chirurgie und Augenheilkunde, von Dr. v. Graefe, &c. 10 band, 3 heft.

*Experiment* 1. One carotid* being tied in a rabbit, produced no peculiar symptom.

*Experiment* 2. Both carotids being tied in a dog, the following symptoms were observed: The action of the heart diminished somewhat; the respiration sunk from forty-two to twenty-eight; weakness of the eyes, sopor, trembling; the body drawn to one side The animal recovered in six days.

*Experiment* 3. Tying both carotids in a rabbit produced no peculiar change.

*Experiment* 4. The same operation on a dog produced sopor, weakness in the eyes, and diminished number of respirations in a minute. In the course of four days the animal recovered.

*Experiment* 5. The same operation in a pigeon produced a very considerable effect: viz. debility of the eyes, vertigo, trembling of the head, inability to stand erect; respiration sinking from 105 to twenty-two in the minute; the temperature diminished 4° 5′ Fahr.; the action of the heart experienced but little change. The animal died on the fourth day, convulsed.

*Experiment* 6. Tying both carotids in a rabbit produced trembling of the head, fæcal vomiting, convulsions, and death in fourteen hours.

*Experiment* 7. The same experiment made on a horse was followed by very violent symptoms: weakness of the eyes, vertigo, collapse as if struck by lightning, incapacity of swallowing, violent madness, convulsions, and death in fifty-eight minutes.

*Experiment* 8 produced the following symptoms in a rabbit: insensibility of parts in opposite directions, viz. the right ear and left eye; expression of alarm; drawing the head to one side; diminution of the heart's action; increased rapidity of respiration. Recovery on the third day.

*Experiment* 9. Tying the two carotids and left par vagum produced the following effects: The head became lifeless, especially upon the left side; the left eye insensible; the animal fell on the left side; convulsions, trismus, and tetanus followed; the respiration became slow and rattling; the heart's action weak, and the temperature fell 15° 75′ Fahr. General paralysis, and death in six hours after the operation. On examination after death, extravasations of blood were found in the lungs and stomach, the lining membrane of which was corroded.

*Experiment* 10. Both carotids of a rabbit were tied; a series of remarkable symptoms were the result. The paralysis was distinctly crucial: there was insensibility of the eye and ear, (the left eye became insensible from the beginning, as also the right ear; whereas, the right eye and left ear continued sensible for some time;) tetanus and trismus; incapacity of swallowing, food returning from the œsophagus; the heart's action but little diminished, whereas the number of respirations fell to sixteen; the temperature sank 27° Fahr.; entire loss of animal heat, and death from loss of sensation and irritability on the fifth day. After death, an unusual quantity of pure gastric juice was found in the stomach. In both eyes an inflammatory membrane had formed on the anterior surface of the iris, closing the pupil.

*Experiment* 11. The symptoms were as follows. Distinct crucial affection of the ear and eye; both eyes afterwards became insensible; tetanus and trismus; the head twisted to one side; chorea; pulsations of the heart reduced to ninety, respiration to twenty; the temperature fell 22° 5′ Fahr. General paralysis, and death in nine hours and a half. Extravasations of blood were found in the stomach, the lining membrane of which was corroded.

*Experiment* 12. Both carotids and the right subclavian artery of a goat being tied, diminished the respiration and action of the heart; it produced affection of the eyes, vertigo, tottering with the head, inability to keep itself on its legs, tetanus, and death in four days.

*Experiment* 13. Injuring the cerebellum of a rabbit, by passing an iron wire through it, and, five days after this, tying the carotids, produced similar symptoms as in the eleventh experiment: the animal died on the eighth day. An inflammatory membrana pupillaris was found in the eye.

* By carotid is understood throughout the Carotis communis.

*Experiment* 14. Injuring the cerebellum of a rabbit produced no peculiar symptom; whereas, tying the carotids seven days after, (in doing which, the right par vagum and sympathetic were included,) produced similar results as in the tenth and eleventh experiments: viz. the right eye was paralyzed, the cornea ulcerated, and, on dissection after death, an effusion of lymph was found on the anterior surface of the iris. The animal died on the twenty-first day.

*Experiment* 15. The two carotids having been tied in a rabbit, the symptoms already described appeared; of which, the fæcal vomiting especially was very severe. An iron pin was passed into the brain on the sixth day: the animal appeared blind, ran against every thing, and died on the eighth day.

*Experiment* 16. Tying the carotid and subclavian arteries in a rabbit produced death in the space of a minute. It was very remarkable, that the heart continued to beat actively six minutes after. Even forty minutes after death, when the animal had become stiff for full ten minutes, the heart still beat eleven times in the minute.

*Experiment* 17. The same operation instantly destroyed a pigeon.

*Experiment* 18. Tying the carotids in a marmot, which was in its hybernating sleep, produced complete rigidity and death.

*Experiments of injecting a foreign Fluid into the Carotids.*

*Experiment* 19. The injection of a little quicksilver into the carotid of a rabbit produced violent suffering, vertigo, twisting of the head, insensibility of the eyes, (first of one, then of the other side,) diminished action of the heart and respiration. The animal died in twenty-two minutes.

*Experiment* 20. Under the same circumstances, lifelessness of the head and body, tetanus, and death in four minutes.

*Experiment* 21. The symptoms were, diminution of the heart's action, of the respiration and animal heat; hemiplegia, and crucial paralysis of the head, eyes, and ears; trismus; refusal to take food; incapacity of swallowing; fæcal vomiting; rigidity, and death on the fourth day. On examining the body, the lining membrane of the stomach was found corroded, and an inflammatory exudation in the eye. The similarity of the phænomena with those observed after tying the carotids is remarkable: (see Exp. 10.)

*Experiment* 22. The paralysis of the head and eye was still more distinct in this instance; the cornea of the left eye ulcerated. Death, on the eighteenth day, resulted from diminution of temperature, rigidity, and want of nourishment.

*Experiment* 23. A small quantity of common injecting wax was thrown into the carotid of a ram. It produced sudden loss of sensibility, of motion, and life, in the head, which spread gradually over the whole body. The respiration stopped, as if the animal seemed to forget the necessity of its continuance. The heart's motion continued active, as if the remaining powers of life had concentrated themselves upon it. The animal died in twelve minutes.

*Experiment* 24. The same phænomena were produced in a goat, and death in eight minutes.

From these experiments Dr. Mayer draws the following conclusions:

1st. That a healthy state of the cerebral activity is the necessary condition of life: in other words, the encephalum (viz. the brain, cerebellum, and medulla oblongata,) is the peculiar source of vital power,—the *fons vitalis.*

2d. The medulla spinalis, of itself, is not sufficient for the continuance of life, as its own life depends upon the vital energy of the encephalum. This inference is deduced especially from the results of the Experiments 23 and 24.

3d. The vertigo and inability of preserving the upright posture is also a result of impeded cerebral activity. We are scarcely justified in attributing these effects to an injured state of the functions of the cerebellum, because, even in the experiments where the vertebral arteries are tied, the cerebellum receives a sufficient supply of blood.

4th. These experiments, moreover, tend to shew that the brain directs and guides the vital functions. The cause or principle of the vital functions (viz. the circulation, respiration, nutrition, animal heat, &c.) is not in the encephalum, but the impulse to exert these functions of vegetative life emanate from it; and they cease when the encephalum sinks into inaction.

The necessity (we might almost say the recollection) of the vital functions stops the instant the vital activity of the encephalum is interrupted: the animal suddenly ceases to breathe, or merely breathes slightly, and always slower; it holds the food in its mouth, forgets to swallow it, or lets it fall out again. The stomach retains some contractile power, but it is an antiperistaltic motion, producing fæcal vomiting. Where the destruction of the activity of the encephalum has been gradual, general insensibility comes on, and the animal dies, from loss of its animal heat, from hunger, rigidity, and exhaustion. If we compare these experiments with those made by Flourens, their results will be found extremely similar. The symptoms which occur in the comatose stage of typhus come under the same head: patients in this state die through insensibility to the vital functions, from paralysis of the encephalum, if they be not continually roused from their sopor, and stimulated by sinapisms, &c.

The physiological researches, especially during the last thirty years, both in this country and the continent, have satisfactorily proved that most, if not all, of the agents which exert such destructive energies on the nervous system do it through the medium of the circulation: this has been shewn by the experiments of Christison and Coindet, of Brodie, Emmert, Viborg, and many others. Those of Sir B. Brodie on the action of the woorara poison are well known. Emmert shewed this to be the case in a still more striking manner, by amputating the leg of an animal, and leaving it connected with the body only by means of the nerves: poisonous substances introduced into the foot produced no effects, not even when applied to the trunk of the nerve; and Viborg even applied one drachm of concentrated prussic acid to the brain of a horse which had been exposed by trepanning, without producing any effect. The experiments of Dupuy* on the contamination of the vital current strikingly confirm Dr. Mayer's observations. He found that injecting water, in which muscle had been soaked for four and a half years, produced symptoms in animals precisely similar to those of typhus; viz. debility, loss of sight, coma, falling of the head, &c. Gaspard† also injected mercury (half an ounce) into the carotid of a sheep, which produced insensibility, coma, and death in fifty minutes; but he does not appear to have carried the subject further in this direction, or to have made any practical deductions from it.

"The impulse," says Professor Mayer, "and the feeling of necessity to keep in action the vital functions, has its seat in the encephalum. Thus, when we reverse the experiment, and separate the head and brain from the trunk, especially in new-born animals, we observe symptoms of the above-mentioned impulse in the decapitated head. The heads of newly-born puppies or kittens, when thus separated from the body, suck the finger which is put into the mouth for ten or fifteen minutes. Attempts at respiration are made by opening the mouth, (a fact first noticed by Le Gallois,) and by the glottis alternately opening and shutting."

Dr. M.'s fifth and last inference is, "that the above-mentioned experiments tend to prove, beyond doubt, that the circulation, the production of animal heat, and lastly nutrition and secretion, depend on the activity of the encephalum, and they stop when it stops; moreover, that the impulse to the continuance of these functions proceeds from the encephalum. It cannot be denied that a variety of causes connected with these functions have their seat in the body; but the main-spring, which sets all the wheels of the vital functions in motion, and without which they stop, resides in the encephalum."

The *anatomical* part of this interesting essay presents some minute research and elaborate investigation, which can only be appreciated properly by aid of the en-

* Injection des Matières putrides dans la Veine Jugulaire du Cheval. Nouv. Bibl. Méd. 1823, Jan., p. 90.

† Mémoire Physiologique sur le Mercure. Magendie's Journal, t. i. No. 2, p. 165.

gravings with which it is illustrated. Our limits, and the nature of the British and Foreign Medical Review, will prevent our taking such copious extracts as we could have wished, and we must merely confine ourselves to a general outline of Professor Mayer's labours.

Experiments have shewn that the most vital part of the encephalum extends from the pons Varolii along the whole medulla oblongata, and at least as far as the second cervical nerve of the spinal marrow; in any part of which a wound is instantly fatal; that, as we descend along the medulla spinalis below this point, or ascend to the brain and cerebellum above it, the effects of injuries become gradually less fatal and dangerous. Professor Mayer's object therefore has been to subject this *centrum vitale* to a minute and rigorous examination, and he has shewn it to be a rich field for observation and discovery. It will be excusable in us to indulge in a little national pride in translating the following passage: if we had no other motive than that of respect for the distinguished physiologist to whom it refers, it would be sufficient; but we deem it no more than fair to shew how honestly and honorably the Germans appreciate the labours of other nations. We give it literally.

"The importance of distinguishing between the anterior and posterior origins of the spinal nerves, since this fact was first established by the talented Charles Bell, has been particularly insisted upon. Magendie has the merit of having fully confirmed these facts by the test of experiment, in which he has so often displayed the master's hand; and Joh. Muller has still more recently confirmed the original views of Bell by experiments on frogs."

The results of Professor Mayer's researches on the origin of the glosso-pharyngeal nerve, the par vagum, the hypoglossus, spinal accessory, and first cervical nerves, is to shew that, of these, the hypoglossus, the spinal accessory, and first cervical, belong to that class of nerves which possess the double faculty of sensation and motion. The question is, does not this compound structure enter more deeply into the organization of the nervous system; or, in other words, shall we not find evidences of it in the phrenic, and even in the sympathetic nerves?

"With regard to the phrenic nerve," says M. Mayer, "I am aware of no researches in which its origin has been traced farther into the fourth cervical nerve. Fig. 1 not only shews the manner in which the phrenic gives off four twigs to the ganglion spinale of the fourth cervical nerve, but is also continued by means of two others along the anterior root of this nerve into the spinal column, so that we may justly say that the phrenic has its origin directly from the medulla spinalis. This is the more remarkable, because the phrenic, with the exception of its connexion with the cardiac nerves of the par vagum and sympathetic, is purely a muscular nerve, (going to the diaphragm). If we examine the sympathetic, we shall find that Soemmering has distinctly mentioned that it receives its roots, or connecting twigs, both from the posterior as also the anterior roots of the spinal nerves."

Professor Mayer has succeeded in tracing the supposed origin of the sympathetic into the spinal marrow itself, not only in animals, but also in the human subject and has given a drawing of the direct and indirect connexion of the sympathetic with the spinal marrow by the anterior and posterior roots of the second lumbar nerve. Fig. 2 shews it in the human subject; and fig. 3 in the calf, to the description of which he refers. According to this, he shews that the sympathetic not only communicates by means of many twigs with the ganglion spinale of the spinal nerves, and thus, with their posterior roots , but that one, two, or even three insulated twigs of the sympathetic are distinctly continued with those of the anterior root into the spinal marrow.

"The nerves," says Professor M., "if I may so express myself, shew a remarkable predilection for decussating or crossing. An arrangement of this kind I have observed in the cardiac nerves; the left branch passing to the right ventricle and pulmonary artery, the right to the left ventricle and aorta. This is not very distinct in the human subject; whereas, in animals it is much more so, and remarkably so in the horse."—"On the other hand, this arrangement of the nervous fibrillæ in the central parts of the nervous system,—viz. the brain and spinal marrow,—is only observed at one spot, viz. at the origin of the corpora pyramidalia. I have always been able to detect this decussation in the human subject, although of variable extent; whereas, in many mammalia, it does not exist at all; in others, again, it appears."

DESCRIPTION OF THE PLATES.

*Figure* 1.

Portion of the spinal cord from which the 3d and 4th cervical nerves arise. Front view, in the human subject.

1. Third cervical nerve. 2. Fourth cervical nerve. 3. Fifth cervical nerve.

*a.* Medulla spinalis.

*b.* Posterior root of the 3d cervical nerve, consisting of five thick cylindrical twigs, and passing into the ganglion spinale.

*c.* Anterior root of the 3d cervical nerve, consisting of three thin flattened bundles with tapering extremities.

*d, d.* Accessory nerve, passing downwards between the two roots of the 3d and 4th cervical nerves.

*e.* Posterior root of the 4th cervical nerve, consisting of two large and thick bundles of filaments, of which the superior gives off a communicating branch to the posterior root of the 3d cervical nerve. They form the ganglion spinale, from which arise

*f.* A cutaneous cervical branch, and

*g.* A muscular branch to the scaleni.

*h.* Anterior root of the 4th cervical nerve. An upper branch is observed arising by three filaments from the spinal cord; it then receives a communicating filament from the lower branch, takes its course to the ganglion spinale, and, passing over it, forms, with another superficial branch, the superior root of

*i.* The phrenic nerve. A lower filament of the anterior root is also seen, which joins the phrenic nerve, as

*k.* The inferior root of the phrenic nerve. Between these two roots of the phrenic nerve *(i, k,)* four slender filaments are observed, which come off from the ganglion spinale.

*l, l.* After this origin, the trunk of the phrenic nerve communicates with the cervical nerve, and then passes downwards.

*Figure* 2.

A portion of the spinal cord from the lumbar region, with the origin of the sympathetic and of the 2d lumbar nerve at this spot, in the human subject.

*a, a.* Portion of the spinal cord from the lumbar region, seen from behind.

*b, b.* Two filaments of the posterior root of the 2d lumbar nerve.

*c.* Ganglion spinale, formed from the above filaments.

*d.* Ramus muscularis dorsalis of the 2d lumbar nerve.

*e.* Anterior branch of the same, (Ramus ileo-inguinalis.)

*f, f.* A part of the lumbar portion of the sympathetic.

*g, h, i.* First, second, and third lumbar ganglion of the sympathetic.

1, 2, 3, 4. Four branches of the anterior root of the 2d lumbar nerve, dissected from each other.

The upper branch (1) takes its course outwards, and, passing by the ganglion, assists to form the branch *d, e.* The branches 2 and 3 unite during their course, but again separate, so that No. 2 passes into the trunk of the nerve, *e,* which is formed of filaments from the ganglion and No. 1. No. 3 joins the sympathetic. The 4th branch passes distinctly by itself into the sympathetic.

*k.* Filament of communication between the 2d lumbar nerve and the sympathetic, dividing into two branches *b* and *m.*

*l.* The upper branch gives off a twig to the nerve *e,* and then passes into the 3d branch of the anterior root of the 2d lumbar nerve.

*m.* The lower branch divides into four filaments; of which, the anterior passes into the nerve, *e,* having previously united with the branch, *l,* by a twig at *n.* Two other filaments unite with the ganglion spinale, and the fourth goes to form the branch No. 4 of the anterior root of the second lumbar nerve.

*Figure* 3.

Portion of the spinal cord at the origin of the second lumbar nerve, in the calf.

*aa.* Anterior surface of the medulla spinalis.

*b, b, b, b, b.* Five branches of the posterior root of the second lumbar nerve.

*c.* Ganglion spinale formed from the above.

*d.* Ramus dorsalis of the second lumbar nerve.

*e.* Ramus anterior of ditto.

*f, f.* Part of the lumbar portion of the sympathetic nerve.

*g.* Second lumbar ganglion of ditto.

*h.* Anterior filament of the anterior root of the second lumbar nerve.

*i.* Branches connected with the lumbar ganglion by two twigs, and continued directly to the spinal cord. Two communicating twigs pass thus to the following branches.

*k.* Second branch of the anterior root of the second lumbar nerve, which receives the two above-mentioned twigs of communication from the former, passing into *d* and *e*.

*l*. Two branches of the sympathetic extending to the ganglion spinale.

*m*. Twelve filaments of the sympathetic, which unite with the trunk *e*.

*Acta Acad. Natur. Curios.* Vol. xvi. Pars II.

## Figures and Tables

**Figure f1:**
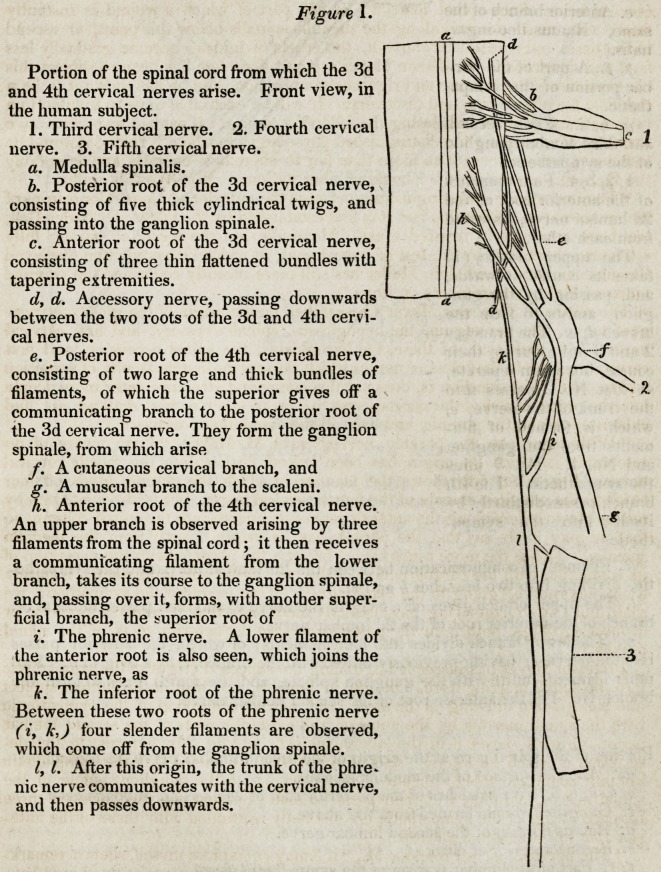


**Figure f2:**
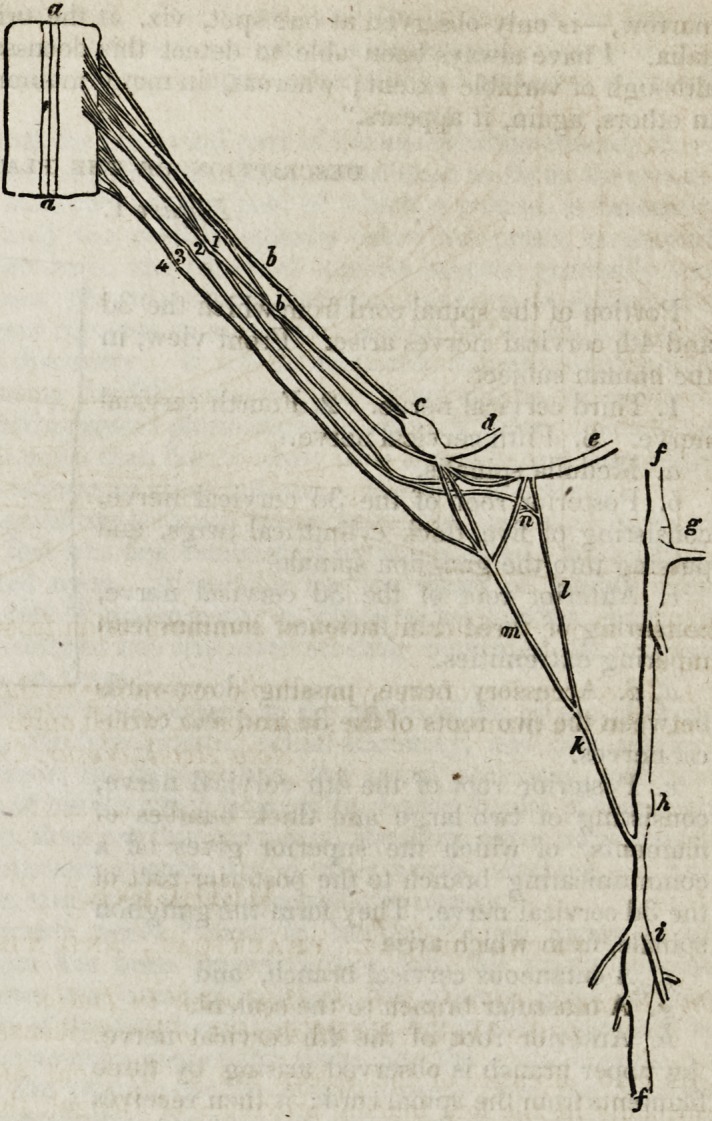


**Figure f3:**